# Effects of Tai Chi on health status in adults with chronic heart failure: A systematic review and meta-analysis

**DOI:** 10.3389/fcvm.2022.953657

**Published:** 2022-09-09

**Authors:** Jiaqi Hui, Ya Wang, Junnan Zhao, Weihong Cong, Fengqin Xu

**Affiliations:** ^1^Laboratory of Cardiovascular Diseases, Xiyuan Hospital, China Academy of Chinese Medical Sciences, Beijing, China; ^2^Institute of Geriatrics, Xiyuan Hospital, China Academy of Chinese Medical Sciences, Beijing, China; ^3^National Clinical Research Center for Chinese Medicine Cardiology, Beijing, China

**Keywords:** Tai Chi, chronic heart failure, health status, systematic review, meta-analysis

## Abstract

**Background:**

Chronic heart failure (CHF) is among the top causes of cardiovascular morbidity, and most patients with CHF have poor health status. Tai Chi, a mind-body exercise that originated in China, is beneficial for health status. This study was conducted to evaluate the effects of Tai Chi on health status in adults with CHF.

**Methods:**

The Cochrane Library, PubMed, Embase, Web of Science, China National Knowledge Infrastructure, Wanfang Database, Chinese Biomedical Database, and Chinese Scientific Journal Database were searched from the inception to 22 October 2021. This meta-analysis was performed using the fixed- or random-effects model. Continuous outcomes were carried out using mean difference (MD) or standardized mean difference (SMD) with 95% confidence interval (CI). Dichotomous outcomes were determined using risk ratio (RR) with 95%CI. The Grading of Recommendations, Assessment, Development and Evaluations (GRADE)pro Guideline Development Tool (GDT) online software was used to present outcome-specific information regarding overall certainty of evidence from studies.

**Results:**

In total, 15 studies including 1,236 participants were finally included. Compared with usual care alone, Tai Chi combined with usual care achieved efficacy in improving Minnesota Living with Heart Failure Questionnaire (MD = −8.51; 95% CI: −10.32 to −6.70; *p* < 0.00001), 6-min walk test (MD = 43.47; 95% CI: 33.38 to 54.10; *p* < 0.00001), left ventricular ejection fraction (MD = 6.07; 95% CI: 3.44 to 8.70; *p* < 0.00001), B-type natriuretic peptide/N-terminal fragment of pro-BNP (SMD = −1.12; 95% CI: −1.70 to −0.54; *p* = 0.0002), Hamilton Depression Rating Scale (MD = −2.89; 95% CI: −4.87 to −0.91; *p* = 0.004), Pittsburgh Sleep Quality Index (MD = −2.25; 95% CI: −3.88 to −0.61; *p* = 0.007), timed up and go test (MD = −1.34; 95% CI: −2.50 to −0.19; *p* = 0.02), and reduced the risk of heart failure hospitalization (RR = 0.47; 95% CI: 0.25 to 0.88; *p* = 0.02). However, there was no difference in the outcome of peak oxygen uptake (MD = 1.38; 95% CI: −1.51 to 4.28; *p* = 0.35). All-cause mortality or cardiovascular death could not be evaluated due to insufficient data. The certainty of evidence ranged from very low to moderate due to the risk of bias, inconsistency, imprecision, and publication bias.

**Conclusion:**

Tai Chi might be safe and showed beneficial effects on health status in patients with CHF. However, more high-quality and long-term studies are still needed to further evaluate the effects of Tai Chi.

## Introduction

Cardiovascular disease (CVD) is the leading cause of mortality and morbidity worldwide (1). Among CVDs, chronic heart failure (CHF) is the terminal manifestation and leading cause of death of CVDs (2). Many patients with CHF live with bothersome symptoms, reduced function, and poor quality of life, which together comprise health status (3–5). The main clinical manifestations among patients with CHF include fatigue, weakness, chest pain at rest or on exertion, dyspnea on exertion, palpitations, reduced physical capacity, mental health problems, and poor quality of sleep. The impaired mental health among patients with CHF is mainly manifested in depression and anxiety, which is associated with five-fold increased mortality risk and increased all-cause readmission (6–8). The symptoms, quality of life, and prognosis are negatively impacted by poor sleep quality in patients with CHF. Initiating and maintaining sleep difficulties (9) dull cognitive processing, impairing information processing, memory, motivation, and decision-making (10). Although guideline-directed therapies are known to improve patient outcomes in CHF (11, 12), few pharmacologic therapies have been demonstrated to improve health status which is associated with subsequent hospitalization and mortality (4).

Tai Chi is a mind-body practice that originated in China as a martial art and has been practiced for health promotion and disease prevention in Asia for many centuries (13, 14). In America, the prevalence of Tai Chi has grown steadily since 2002, with a 14.5% prevalence in 2017. Tai Chi has increased in each demographic across the United States. Most people report using Tai Chi for overall wellness; however, approximately 15% of people use Tai Chi to help with specific medical conditions (15, 16). Tai Chi combines meditation with slow, gentle, graceful movements, as well as deep breathing and relaxation, to move vital energy (or qi) throughout the body (13). It has developed into various kinds of styles or forms during its evolution, including Chen-, Wu-, Sun-, and Yang-style with 24 forms or 42 forms (17, 18) which is considered a complex, multicomponent intervention that integrates physical, psychosocial, emotional, spiritual, and behavioral elements (13, 19). Previous research and meta-analyses have suggested that Tai Chi offers a therapeutic benefit in patients with CHF (17, 20–23). However, mental health and sleep quality as important components of health status were not concerned in the previous meta-analyses, so we found new outcomes to evaluate the effects of Tai Chi on patients with CHF, including Hamilton Depression Rating Scale (HAMD) and Pittsburgh Sleep Quality Index (PSQI). Moreover, heart failure hospitalization, all-cause mortality or cardiovascular death, and adverse events were also assessed to evaluate the effects of Tai Chi on long-term cardiovascular prognosis in patients with CHF. In addition, the evidence of Tai Chi for CHF was assessed in Huang et al.’s study (24). The generally low quality of six systematic reviews and meta-analyses and outcome indicators meant that it was impossible to draw firm conclusions about Tai Chi. Therefore, to determine the effect of Tai Chi on CHF, it was necessary to more comprehensively evaluate the outcome indicators and carry out this systematic review more rigorously.

## Methods

This systematic review and meta-analysis were reported according to the Preferred Reporting Items for Systematic Reviews and Meta-Analyses (PRISMA) reporting guidelines (25), and the PRISMA checklist is provided in Supplementary Table 1. The protocol was previously registered in the International Prospective Register of Systematic Reviews, PROSPERO, under CRD42022309754. Since this study is a systematic review and meta-analysis of human intervention studies, it did not require approval from an ethical committee.

### Search strategy

Electronic databases including the Cochrane Library, PubMed, Embase, Web of Science, China National Knowledge Infrastructure (CNKI), Wanfang Database, Chinese Biomedical Database (SinoMed), and Chinese Scientific Journal Database (Chinese VIP Information) were searched to identify all relevant studies. We also conducted a gray literature search using existing literature review records and reference list of identified studies. The electronic search was supplemented by hand-searching reference list of previous systematic reviews. No language or race restrictions were applied. The date was restricted from the inception to 22 October 2021. The Medical Subject Heading and the following free words were searched including “Tai Ji,” “Tai Chi,” “Chi, Tai,” “Tai Ji Quan,” “Heart Failure,” “Cardiac Failure,” “Heart Decompensation,” etc. The retrieval expressions were formed by logically connecting AND or OR. The full search strategy is shown in Supplementary Table 2.

### Selection criteria

The inclusion criteria were as follows: (1) patients were all adults aged 18 years or older with a diagnosis of CHF (all types); (2) any form of Tai Chi was used as a part of clinical interventions lasting for at least 3 months; (3) usual care or cardiac rehabilitation was carried out in the control group, such as health education, pharmacologic therapy, dietary, aerobic exercise, and endurance training; (4) primary outcomes including Minnesota Living with Heart Failure Questionnaire (MLHFQ), 6-min walk test (6MWT), and left ventricular ejection fraction (LVEF); secondary outcomes including B-type natriuretic peptide (BNP) or N-terminal fragment of pro-BNP (NT-pro-BNP), HAMD, PSQI, peak oxygen uptake (peak VO_2_), timed up and go test (TUGT), heart failure hospitalization, all-cause mortality or cardiovascular death, and adverse events; (5) randomized controlled trials (RCTs) were delivered in a complete paper article.

The exclusion criteria were as follows: (1) patients in the control group performed physical activities originating in China, such as Qigong, and Baduanjin; (2) the Tai Chi group included other physical activities originating in China except for Tai Chi; (3) the intervention period was less than 3 months; (4) incomplete data, duplicate data, no extractable data or no relevant outcomes were reported; (5) the types of studies were reviews, commentaries, case reports, conference abstracts with incomplete data, retrospective studies, etc.

### Study selection and data extraction

Study selection was carried out with the EndNote X9. All study records identified in the search were downloaded and duplicates were identified and deleted. Afterward, two review authors (J. Hui and Y. Wang), working in pairs, independently screened titles and abstracts (step 1) and then full text (step 2) of potentially relevant records. A third review author resolved any disagreements between the two review authors. The study selection was documented in a flow chart in the systematic review, as per PRISMA guidelines. A standard data extraction form was developed and trialed until data extractors reached convergence and agreement. In total, two review authors independently extracted data on study characteristics in a standardized manner, including study authors, study year, characteristics of participants (e.g., age, gender, sample size, diagnosis, New York Heart Association (NYHA) class, LVEF), details of the Tai Chi group (e.g., the form of Tai Chi, frequency, duration) and the control group (e.g., health education, pharmacologic therapy, dietary, aerobic exercise, endurance training), and outcomes. If there was any unclear or missing information, the original authors were contacted for additional information or clarification where required. If the literature had multiple endpoint indicators, the longest one was adopted. A third review author resolved the conflicts in data extraction.

### Quality assessment

The risk of bias in selected studies was independently assessed by two review authors (J. Hui and Y. Wang), under the guidance of the Cochrane Collaboration’s Risk of Bias Handbook for RCTs. In total, seven domains were used to assess the methodological quality, including random sequence generation (selection bias), allocation concealment (selection bias), blinding of participants and personnel (performance bias), blinding of outcome assessment (detection bias), incomplete outcome data (attrition bias), selective reporting (reporting bias), and other bias. Assessment of each domain was classified as “low risk of bias,” “high risk of bias,” or “unclear risk of bias” in accordance with the recommendations of the Cochrane Handbook. Any disagreement was resolved by a third review author. To confirm and validate the methods of allocation concealment and the randomization procedure, the original authors were contacted. If the original authors did not get in touch, any disagreements were resolved through discussion. Furthermore, overall quality of included evidence was deemed very low, low, moderate, or high using the Grading of Recommendations, Assessment, Development and Evaluations (GRADE)pro Guideline Development Tool (GDT) online software, which included total number of studies, study design, risk of bias, inconsistency, indirectness, imprecision, other considerations, summary of findings, and importance.

### Data synthesis and analysis

Statistical analysis was performed using Review Manager 5.3 software. Continuous outcomes (including MLHFQ, 6MWT, LVEF, BNP/NT-pro-BNP, HAMD, PSQI, peak VO_2_, and TUGT) were carried out using mean difference (MD) or standardized mean difference (SMD) with 95% confidence interval (CI). MD was used when the outcome was carried out using the same methodology; otherwise, SMD was used instead. The mean changes (pre–post-intervention) and the standard deviations (SDs) of the mean changes were inputted into Review Manager 5.3 software to evaluate the outcomes for the Tai Chi group and the control group. Dichotomous outcomes (including heart failure hospitalization, all-cause mortality or cardiovascular death, and adverse events) were determined using risk ratio (RR) with 95% CI. Two or more clinically homogenous studies were found to be sufficiently homogenous statistically to be combined in a meta-analysis. Statistical heterogeneity was tested using the Chi^2^ (χ^2^) test and *I*^2^ statistic. Studies with an *I*^2^ statistic of 25–50% were considered to reflect low heterogeneity, those with an *I*^2^ statistic of 50–75% were considered to reflect moderate heterogeneity, and those with an *I*^2^ statistic of >75% were considered to reflect high heterogeneity. A fixed-effects model was used in low heterogeneity (*I*^2^ ≤ 50%), and a random-effects model was used for higher heterogeneity (*I*^2^ > 50%). All reported *p*-values were two-tailed and considered statistically significant when *p* < 0.05. When heterogeneity was present, subgroup analyses were performed based on different treatment courses (3, 6, and 12 months) to explore the sources of significant heterogeneity of the results. Sensitivity analyses, which estimated the robustness of the results, were also conducted to assess the impact of each study on the overall effect size by excluding studies one by one from the meta-analysis.

### Publication bias

Publication bias was unable to formally assess whether there were fewer than 10 included studies. Publication bias was evaluated through visual inspection of funnel plot with Stata 12.0 software, and asymmetry suggested publication bias. Meanwhile, the Egger’s test was performed to provide statistical evidence of funnel plot asymmetry. If *p* > 0.05, there is no publication bias and vice versa. The Duval and Tweedie “trim and fill” method was used to adjust the analysis for the effects of publication bias.

## Results

### Study selection

**FIGURE 1:**
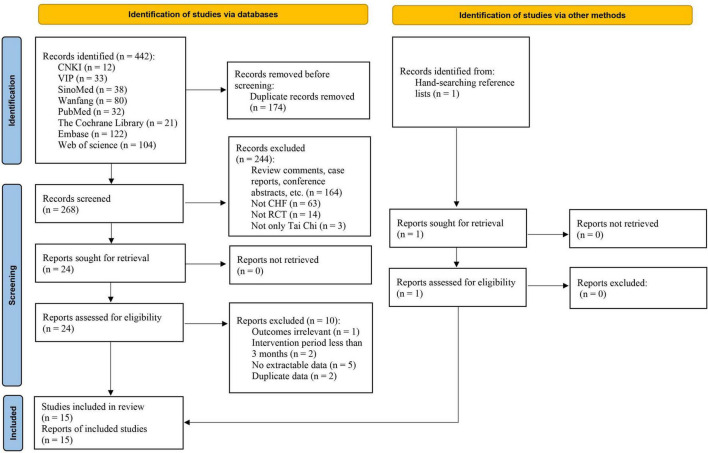
Flow diagram of the study selection process. CHF, chronic heart failure; CNKI, China National Knowledge Infrastructure; RCT, randomized controlled trial; SinoMed, Chinese Biomedical Database; VIP, Chinese Scientific Journal Database (Chinese VIP Information).

The literature searching process and study identification are summarized in Figure 1. Searches of the listed databases using the search string identified 442 potential articles, and 268 records remained for first-stage screening after the exclusion of the duplicate records. Further screening of titles and abstracts excluded 244 records, mainly because they were irrelevant to the aim of the study. For the 24 records that underwent full-text review, 10 were excluded because 1 of them did not report the relevant outcomes of the study, 2 reported the intervention period less than 3 months, 5 included no extractable data, and the other 2 reported duplicate data. For the 10 excluded studies that most closely resembled inclusion criteria, the reasons for exclusion are listed in Supplementary Table 3. Besides, 1 study (26) was identified by hand-searching reference list of previous systematic reviews.

### Study characteristics

Overall, 15 RCTs were included. The baseline characteristics of the included RCTs are reported in Table 1. The studies were published between 2004 and 2021. There were a total of 1236 participants included in the studies. The mean age of patients with CHF was 64.05 ± 9.96 years, with NYHA classes ranging from I to IV. States or regions of participants were mainly from China (*n* = 12, 80%), the United States (*n* = 2, 13.3%), and Italy (*n* = 1, 6.7%). The population included in the meta-analysis was homogenous in terms of their demographics and clinical characteristics. Sample sizes of the included studies ranged from 16 to 200, with 644 patients in the Tai Chi group and 592 patients in the control group. Tai Chi was performed in different forms or styles, including 24-form (*n* = 8, 53.3%) (27–34), 42-form (*n* = 1, 6.7%) (35), Yang-style (*n* = 4, 26.7%) (26, 31, 36, 37), and Chen-style (*n* = 2, 13.3%) (35, 38). However, 2 studies (13.3%) (39, 40) did not specify the form or style of Tai Chi. Exercise time lasted 15–60 min every time, the frequency of Tai Chi varied from two times to seven times per week, and the duration of Tai Chi ranged from 3 to 12 months. Usual care or cardiac rehabilitation included health education, pharmacologic therapy (diuretics, vasodilators, angiotensin-converting enzyme inhibitors, beta-blockers, statins, etc.), dietary, aerobic exercise, endurance training, etc.

**TABLE 1:** Characteristics of the 15 trials included in the systematic review and meta-analysis.

References	Country	Age (mean ± SD, year)	Gender (male/female)	Sample size (Tai Chi/Control)	Participants	NYHA class	LVEF (mean ± SD, %)	Intervention: Tai Chi group				Control group	Outcomes
								Form/Style	Frequency	Duration (month)	Total exercise time per week		
Caminiti et al. (26)	Italy	>65 (73.8 ± 6)	51/9	60 (30/30)	CHF due to left ventricular systolic dysfunction	II	<45 (33 ± 9)	A modified 10-movement Yang style (10 min of warm-up exercises, 30 min of Tai Chi practice, and 10 min of cool-down exercises)	1 h/time, 4 times/week	3	240 min	Endurance training (10 min of warm-up, 10 min of cool-down and flexibility exercises, and 30 min of aerobic exercise with cycling or walking at 60–70% of estimated VO_2_ max)	6MWT, NT-pro-BNP
Ding and Chen (38)	China	50–69 (61.4 ± 5.2)	19/11	60 (30/30)	CHF	II or III	Not report	Chen-style of Tai Chi, usual care: Qili qiangxin capsules (4 pills, t.i.d., p.o.), acupressure, and dietary.	30 min/session, 2 sessions/day, 7 days/week	3	420 min	Usual care: Qili qiangxin capsules (4 pills, t.i.d., p.o.), acupressure, and dietary.	MLHFQ, 6MWT
Liu (27)	China	35–65 (54.98 ± 7.45)	31/35	66 (33/33)	CHF, CHD	II or III	44.91 ± 0.59	Simplified 24 forms of Tai Chi, pharmacologic therapy (diuretics, vasodilators, angiotensin-converting enzyme inhibitors, beta-blockers, etc.)	1 h/time, 3–4 times/week	3	240 min	Pharmacologic therapy (diuretics, vasodilators, angiotensin-converting enzyme inhibitors, beta-blockers, etc.)	MLHFQ, 6MWT, LVEF
Pan (28)	China	61–82 (66.96 ± 11.88)	35/26	61 (31/30)	CHF	II or III	≤45 (32.74 ± 7.25)	Simplified 24 forms of Tai Chi, usual care: dietary, health education, pharmacologic therapy (diuretics, angiotensin-converting enzyme inhibitors, beta-blockers, etc.)	≥30 min/day, 7 days/week	6	210 min	Usual care: dietary, health education, pharmacologic therapy (diuretics, angiotensin-converting enzyme inhibitors, beta-blockers, etc.)	LVEF, 6MWT, BNP
Sang et al. (39)	China	61–80 (70.75 ± 9.55)	57/43	100 (50/50)	CHF, CHD	II or III	36.7 ± 2.9	Tai Chi rehabilitation, pharmacologic therapy	15 min/day, 7 days/week	3	105 min	Pharmacologic therapy	MLHFQ, 6MWT, LVEF
Sang et al. (40)	China	60–77 (65.75 ± 5.83)	35/25	60 (30/30)	CHF, CHD	II or III	35.95 ± 2.89	Tai Chi rehabilitation, pharmacologic therapy	15 min/day, 7 days/week	3	105 min	Pharmacologic therapy	LVEF, BNP
Wang (29)	China	63–71 (66.86 ± 1.86)	23/33	56 (28/28)	CHF	I, II or III	Not report	Simplified 24 forms of Tai Chi, pharmacologic therapy (angiotensin-converting enzyme inhibitors, beta-blockers, etc.)	30 min/time, 5 times/week	3	150 min	Walking training, pharmacologic therapy (angiotensin-converting enzyme inhibitors, beta-blockers, etc.)	6MWT
Yang et al. (30)	China	>65 (70.65 ± 5.88)	52/42	94 (47/47)	CHF	I, II or III	51.38 ± 2.95	Simplified 24 forms of Tai Chi, pharmacologic therapy (diuretics, angiotensin-converting enzyme inhibitors, statins, etc.)	1 h/time, 6 times/week	12	360 min	Pharmacologic therapy (diuretics, angiotensin-converting enzyme inhibitors, etc.)	6MWT, TUGT, NT-pro-BNP, LVEF
Yao et al. (35)	China	52.07 ± 6.76	89/61	150 (80/70)	CHF	II	30.56 ± 9.54	Chen-style 42 forms of Tai Chi, lifestyle change, dietary, pharmacologic therapy (diuretics, vasodilators, beta-blockers, etc.)	≥30 min/time, 5 times/week	6	150 min	Lifestyle change, dietary, pharmacologic therapy (diuretics, vasodilators, beta-blockers, etc.)	MLHFQ, 6MWT, LVEF
Yeh et al. (36)	The United States	64 ± 13	19/11	30 (15/15)	CHF	I, II, III or IV	≤40 (23 ± 7)	The five simplified Tai Chi movements adapted from Master Cheng Man-Ch’ing’s Yang-style short form, usual care: pharmacologic therapy (angiotensin-converting enzyme inhibitor, beta-blocker, loop diuretic, digoxin, spironolactone, etc.), dietary and exercise counseling	1 h/time, two times/week	3	120 min	Usual care: pharmacologic therapy (angiotensin-converting enzyme inhibitor, beta-blocker, loop diuretic, digoxin, spironolactone, etc.), dietary and exercise counseling	MLHFQ, 6MWT, BNP, peak VO_2_, all-cause death
Yeh et al. (37)	The United States	66 ± 12	8/8	16 (8/8)	CHF	I, II or III	≥50 (63.5 ± 8.37)	The five simplified Tai Chi movements adapted from Master Cheng Man-Ch’ing’s Yang-style short form	1 h/time, two times/week	3	120 min	Aerobic exercise control	MLHFQ, LVEF, BNP, 6MWT, peak VO_2_, TUGT, adverse events, hospitalizations
Yu et al. (31)	China	18–75 (59.5 ± 11.9)	73/47	120 (80/40)	CHF	I, II or III	≤45 (31.5 ± 8.55)	Yang-style simplified 24 forms of Tai Chi, pharmacologic therapy (angiotensin-converting enzyme inhibitor, angiotensin II receptor blocker, beta-blocker, loop diuretic, spironolactone, etc.)	30/60 min/time, 5 times/week	6	300 min	Health education, walking training (30 min), pharmacologic therapy (angiotensin-converting enzyme inhibitor, angiotensin II receptor blocker, beta-blocker, loop diuretic, spironolactone, etc.)	6MWT, BNP, LVEF
Yu et al. (32)	China	≥60 (68 ± 6.1)	106/94	200 (100/100)	CHF after myocardial infarction	II or III	≥45 (46.45 ± 3.895)	Simplified 24 forms of Tai Chi, health education, pharmacologic therapy (diuretics, angiotensin-converting enzyme inhibitors, beta-blockers, etc.)	20 min/time, two times a day, 7 days/week	6	280 min	Health education, pharmacologic therapy (diuretics, angiotensin-converting enzyme inhibitors, beta-blockers, etc.)	BNP, LVEF, 6MWT, cardiovascular death, hospitalization, MLHFQ
Yuan (33)	China	60–80 (66.9 ± 4.76)	33/27	60 (30/30)	CHF	II or III	≤40 or ≥50 (42.95 ± 3.42)	Simplified 24 forms of Tai Chi, pharmacologic therapy (diuretics, angiotensin-converting enzyme inhibitors, beta-blockers, etc.)	15–40 min/time, 3–5 times/week	3	200 min	Pharmacologic therapy (diuretics, angiotensin-converting enzyme inhibitors, beta-blockers, etc.)	BNP, LVEF, 6MWT, HAMD, PSQI, MLHFQ
Zhou et al. (34)	China	55–69 (62.1 ± 5.78)	57/46	103 (52/51)	CHF	II or III	≤45 (41.13 ± 4.99)	24 forms of Tai Chi, cardiac rehabilitation and exercise training	15–40 min/time, 5 times/week	3	200 min	Cardiac rehabilitation and exercise training	LVEF, HAMD, PSQI, MLHFQ

CHD, coronary heart disease; CHF, chronic heart failure; BNP, B-type natriuretic peptide; HAMD, Hamilton Depression Rating Scale; LVEF, left ventricular ejection fraction; MLHFQ, Minnesota Living with Heart Failure Questionnaire; NT-pro-BNP, N-terminal fragment of pro-BNP; NYHA, New York Heart Association; Peak VO_2_, peak oxygen uptake; PSQI, Pittsburgh Sleep Quality Index; SD, standard deviations; TUGT, timed up and go test; 6MWT, 6-min walk test.

### Risk of bias

**FIGURE 2:**
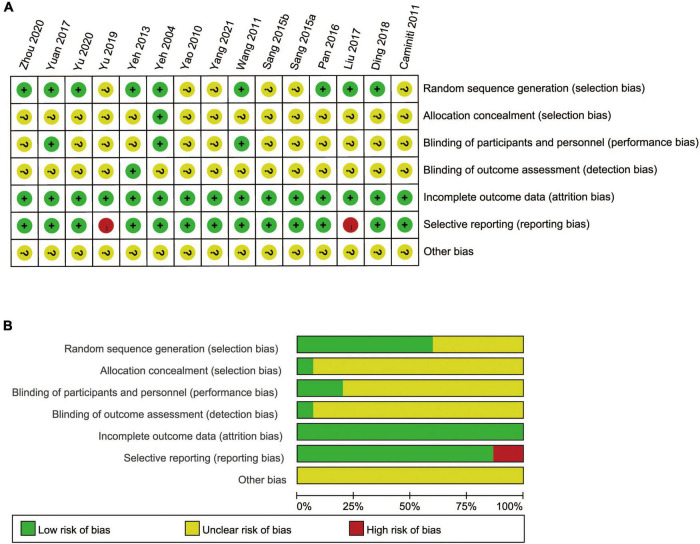
Risk of bias summary **(A)** and bias graph **(B)**.

The risk of bias of the included 15 RCTs is shown in Figure 2. Among 15 studies, 6 trials (40%) presented an unclear risk of bias in the sequence generation process, 1 trial (6.7%) reported the opaque envelopes to achieve allocation concealment, and 14 trials (93.3%) presented an unclear selection risk in allocation concealment. In total, twelve studies (80%) obtained an unclear risk of performance bias in blinding of outcome assessment due to the characteristic of Tai Chi, which was difficult to perform blinding. Only one study (6.7%) presented a low risk of detection bias in blinding of outcome assessment, whereas 14 trials (93.3%) presented an unclear risk of bias. All trials (100%) included complete outcome data and presented a low risk of attrition bias. In terms of selective reporting bias, 13 trials (86.7%) reported all the outcomes listed in the methods section and presented a low risk of bias, whereas 2 trials (13.3%) presented a high risk of bias. We did not identify any other source of bias in any of the included studies and considered all studies unclear risk of bias. Besides, among 15 RCTs, 3 studies (20%) reported multiple funding sources, 5 studies (33.3%) were also funded and the funding sources of each study were governmental agencies, and the remaining 7 trials (46.7%) did not disclose any sources of funding. These studies were judged as probably low risk of bias for conflict of interests. There was no specific funding for the meta-analysis, and more details about the funding sources are listed in Supplementary Table 4. In brief, the quality of the included RCTs was relatively not high, thus weakening the strength and trustworthiness of the clinical evidence of Tai Chi.

### Primary outcomes

#### Minnesota Living with Heart Failure Questionnaire (Score)

**FIGURE 3:**
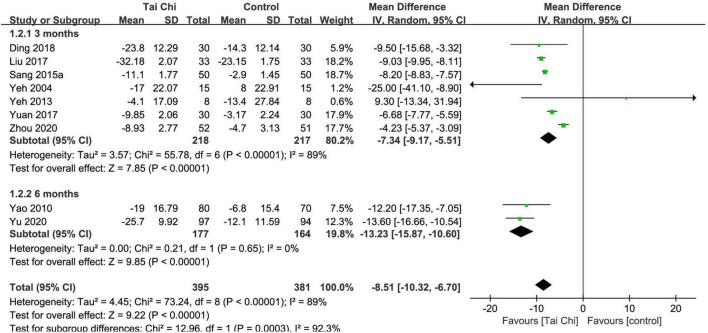
Meta-analysis of MLHFQ in the Tai Chi group vs. the control group. CI, confidence interval; IV, inverse variance; SD, standard deviation.

In total, nine trials reported the effect of Tai Chi on MLHFQ. Meta-analysis showed that MLHFQ scores were significantly reduced in the Tai Chi group compared with the control group (MD = −8.51; 95% CI: −10.32 to −6.70; *p* < 0.00001), but represented statistical heterogeneity (χ^2^ = 73.24; *I*^2^ = 89%) with the random-effects model. The leave-1-out sensitivity analysis showed that the results were robust. The results were statistically significant in the subgroup analysis after the 3- and 6-month intervention between the Tai Chi and the control group (Figure 3).

#### 6-min walk test (m)

**FIGURE 4:**
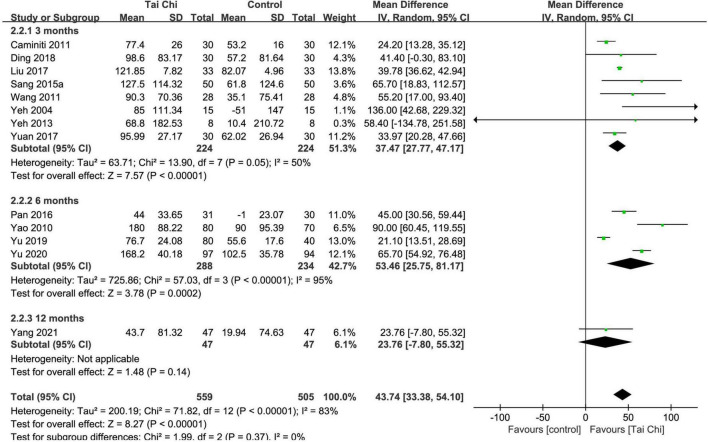
Meta-analysis of 6MWT in the Tai Chi group vs. the control group. CI, confidence interval; IV, inverse variance; SD, standard deviation.

In total, 13 trials reported the effect of Tai Chi on 6MWT. Meta-analysis showed that 6MWT was significantly improved in the Tai Chi group compared with the control group (MD = 43.47; 95% CI: 33.38 to 54.10; *p* < 0.00001), but represented statistical heterogeneity (χ^2^ = 71.82; *I*^2^ = 83%) with the random-effects model. The results were statistically significant in the subgroup analysis after the 3- and 6-month intervention, but in the subgroup analysis after the 12-month intervention, there was no statistically significant difference between the Tai Chi and the control group (Figure 4). Sensitivity analyses showed that the results were robust for 6MWT.

#### Left ventricular ejection fraction (%)

**FIGURE 5:**
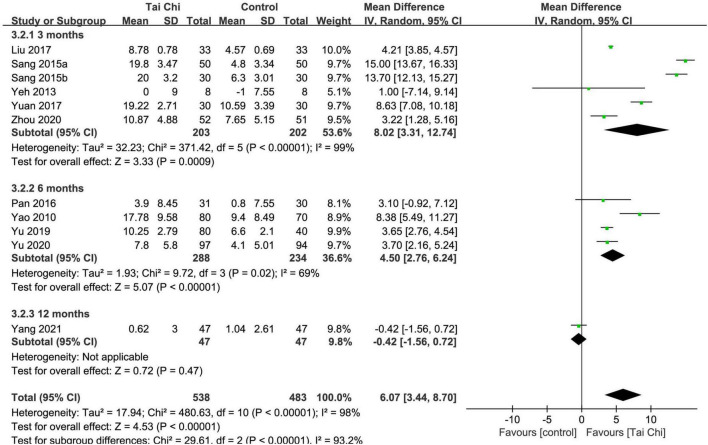
Meta-analysis of LVEF in the Tai Chi group vs. the control group. CI, confidence interval; IV, inverse variance; SD, standard deviation.

In total, eleven trials reported the effect of Tai Chi on LVEF. Absolute LVEF values before and after treatment are presented in Supplementary Table 5. Meta-analysis showed that LVEF was significantly improved in the Tai Chi group compared with the control group (MD = 6.07; 95% CI: 3.44 to 8.70; *p* < 0.00001), but represented statistical heterogeneity (χ^2^ = 480.63; *I*^2^ = 98%) with the random-effects model. The results were statistically significant in the subgroup analysis after the 3- and 6-month intervention, but in the subgroup analysis after the 12-month intervention, there was no statistically significant difference between the Tai Chi and the control group (Figure 5). Sensitivity analyses showed that the results were robust for LVEF.

### Secondary outcomes

#### B-type natriuretic peptide/N-terminal fragment of pro-B-type natriuretic peptide (pg/ml)

**FIGURE 6:**
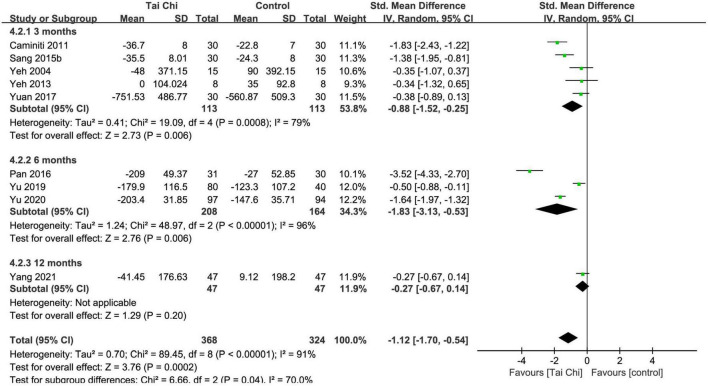
Meta-analysis of BNP/NT-pro-BNP in the Tai Chi group vs. the control group. CI, confidence interval; IV, inverse variance; SD, standard deviation.

In total, nine trials reported the two types of this outcome with different standards, which are listed in Supplementary Table 6, so SMD was chosen as the result. Meta-analysis showed that BNP/NT-pro-BNP was significantly improved in the Tai Chi group compared with the control group (SMD = −1.12; 95% CI: −1.70 to −0.54; *p* = 0.0002), but represented statistical heterogeneity (χ^2^ = 89.45; *I*^2^ = 91%) with the random-effects model. The results were statistically significant in the subgroup analysis after the 3- and 6-month intervention, but there was no statistically significant difference between the Tai Chi and the control group in the subgroup analysis after the 12-month intervention (Figure 6). Sensitivity analyses showed that the results were robust for BNP/NT-pro-BNP.

#### Hamilton Depression Rating Scale (Score)

**FIGURE 7:**
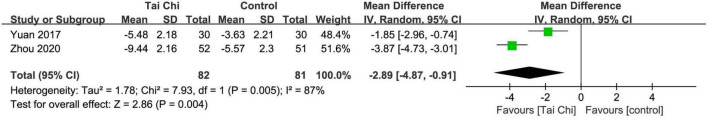
Meta-analysis of HAMD in the Tai Chi group vs. the control group. CI, confidence interval; IV, inverse variance; SD, standard deviation.

In total, two trials reported the effect of Tai Chi on depression. HAMD scores were significantly reduced in the Tai Chi group compared with the control group (MD = −2.89; 95% CI: −4.87 to −0.91; *p* = 0.004). There was significant heterogeneity (χ^2^ = 7.93; *I*^2^ = 87%) and the random-effects model was used (Figure 7). Sensitivity analysis was not conducted because of insufficient numbers of included trials.

#### Pittsburgh Sleep Quality Index (Score)

**FIGURE 8:**
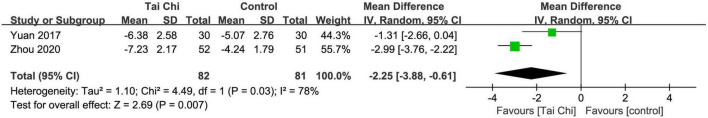
Meta-analysis of PSQI in the Tai Chi group vs. the control group. CI, confidence interval; IV, inverse variance; SD, standard deviation.

In total, two trials reported the effect of Tai Chi on sleep quality. PSQI scores were significantly reduced in the Tai Chi group compared with the control group (MD = −2.25; 95% CI: −3.88 to −0.61; *p* = 0.007). There was significant heterogeneity (χ^2^ = 4.49; *I*^2^ = 78%) and the random-effects model was used (Figure 8). Sensitivity analysis was not conducted because of insufficient numbers of included trials.

#### Peak oxygen uptake (ml/kg/min)

**FIGURE 9:**
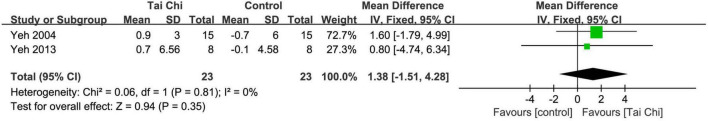
Meta-analysis of peak VO2 in the Tai Chi group vs. the control group. CI, confidence interval; IV, inverse variance; SD, standard deviation.

In total, two trials showed no statistically significant difference in improving peak VO_2_ between the Tai Chi and the control group (MD = 1.38; 95% CI: −1.51 to 4.28; *p* = 0.35). There was no evidence of heterogeneity (χ^2^ = 0.06; *I*^2^ = 0%) and the fixed-effects model was used (Figure 9). Sensitivity analysis was not conducted because of insufficient numbers of included trials.

#### Timed up and go test (s)

**FIGURE 10:**
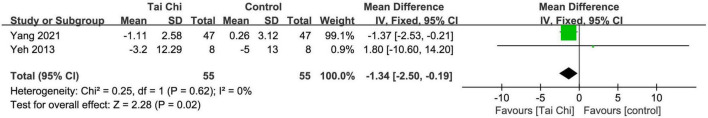
Meta-analysis of TUGT in the Tai Chi group vs. the control group. CI, confidence interval; IV, inverse variance; SD, standard deviation.

In total, two trials demonstrated that Tai Chi was effective in decreasing the time of TUGT (MD = −1.34; 95% CI: −2.50 to −0.19; *p* = 0.02) with no evidence of heterogeneity (χ^2^ = 0.25; *I*^2^ = 0%, Figure 10). The fixed-effects model was used. Sensitivity analysis was not conducted because of insufficient numbers of included trials.

### Heart failure hospitalization

**FIGURE 11:**
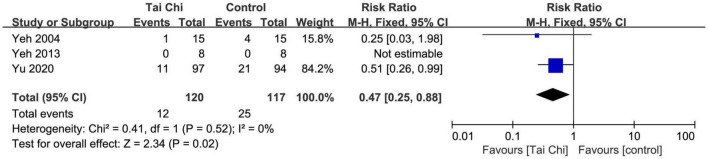
Meta-analysis of heart failure hospitalization in the Tai Chi group vs. the control group. CI, confidence interval.

In total, three trials reported this outcome. The meta-analysis showed that Tai Chi was effective in reducing the risk of heart failure hospitalization compared with the control group (RR = 0.47; 95% CI: 0.25 to 0.88; *p* = 0.02). There was no evidence of heterogeneity (χ^2^ = 0.41; *I*^2^ = 0%) and the fixed-effects model was used (Figure 11). Sensitivity analysis was not conducted because of insufficient numbers of included trials.

### All-cause mortality or cardiovascular death

In total, two trials reported this outcome. The effects of Tai Chi on all-cause mortality or cardiovascular death could not be evaluated due to insufficient data. One study (32) reported that 3 (3%) patients were dead due to cardiovascular events in the Tai Chi group and 6 (6%) in the control group. However, the other study reported that there were no deaths during the study period in either group (36).

### Adverse events

In total, two trials (36, 37) reported this outcome. No adverse event occurred during the study period between the Tai Chi and the control group.

### Publication bias

**FIGURE 12:**
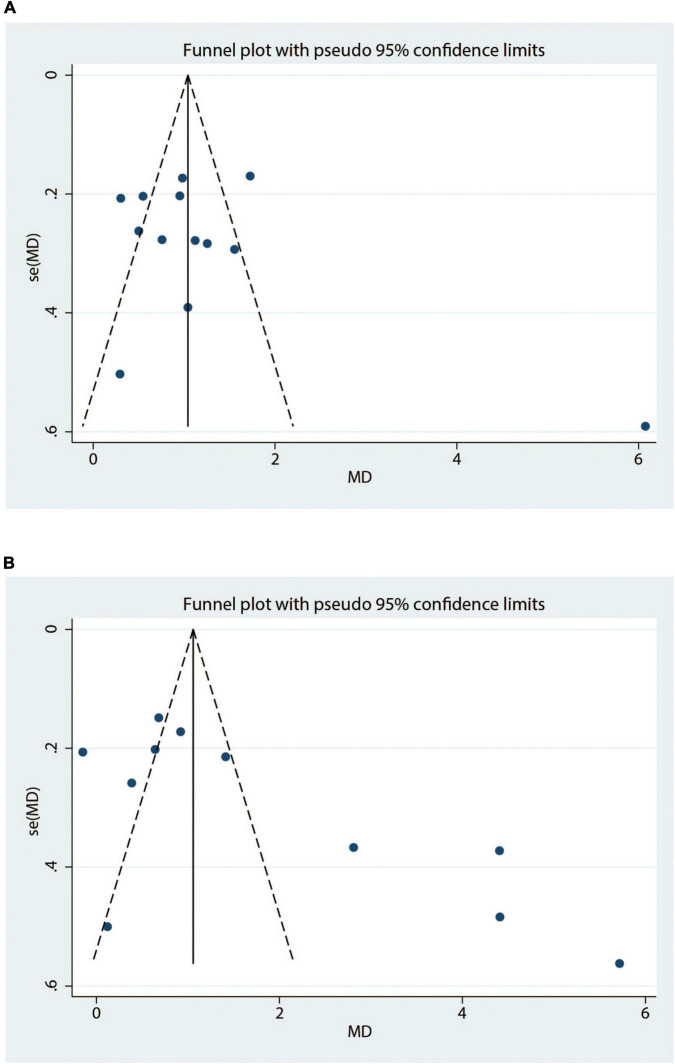
Funnel plots of 6MWT **(A)** and LVEF **(B)**. MD, mean difference.

Publication biases for 6MWT (Figure 12A) and LVEF (Figure 12B) were assessed through visualization of funnel plots. The asymmetrical funnel plots suggested high risk of publication bias. Both funnel plot and Egger’s test showed no risk of publication bias in 6MWT, while high risk in LVEF. After conducting the “trim and fill” method, publication bias had no significant impact on meta-analysis results (Table 2).

**TABLE 2:** The results of publication bias and “trim and fill” test.

Outcomes	Egger’s test		“Trim and fill” test
	*T*	*P*	
6MWT	1.14	0.28	/
LVEF	2.91	0.017	(MD = 1.87; 95% CI: 1.084 to 2.657; *p* = 0.000 < 0.05)

CI, confidence interval; LVEF, left ventricular ejection fraction; MD, mean difference; 6MWT, 6-min walk test.

### Quality of the evidence

All of the outcomes of interest were assessed with GRADEpro GDT and the evidence profile is shown in Table 3. There was moderate-quality evidence on TUGT and heart failure hospitalization; low-quality evidence on MLHFQ, 6MWT, BNP/NT-pro-BNP, HAMD, PSQI, peak VO_2_, and all-cause mortality or cardiovascular death; and very low-quality evidence on LVEF. The certainty of the evidence was not high, which might be attributed to the risk of bias, inconsistency, imprecision, and publication bias, indicating that these estimates were uncertain and future studies might influence our confidence in the results.

**TABLE 3:** The GRADE evidence profile for Tai Chi in the treatment of patients with CHF.

Certainty assessment							No. of patients		Effect		Certainty	Importance
No. of studies	Study design	Risk of bias	Inconsistency	Indirectness	Imprecision	Other considerations	[intervention]	[comparison]	Relative (95% CI)	Absolute (95% CI)		
**MLHFQ**												
9	Randomized trials	Serious^a^	Serious^b^	Not serious	Not serious	None^d^	395	381	–	MD 8.51 lower (10.32 lower to 6.7 lower)	⊕⊕○○ Low	IMPORTANT
**6MWT**												
13	Randomized trials	Serious^a^	Serious^b^	Not serious	Not serious	None	559	505	–	MD 43.74 higher (33.38 higher to 54.1 higher)	⊕⊕○○ Low	IMPORTANT
**LVEF**												
11	Randomized trials	Serious^a^	Serious^b^	Not serious	Not serious	Publication bias strongly suspected	538	483	–	MD 6.07 higher (3.44 higher to 8.7 higher)	⊕○○○ Very low	IMPORTANT
**BNP/NT-pro-BNP**												
9	Randomized trials	Serious^a^	Serious^b^	Not serious	Not serious	None^d^	368	324	–	SMD 1.12 lower (1.7 lower to 0.54 lower)	⊕⊕○○ Low	IMPORTANT
**HAMD**												
2	Randomized trials	Serious^a^	Serious^b^	Not serious	Not serious	None^d^	82	81	–	MD 2.89 lower (4.87 lower to 0.91 lower)	⊕⊕○○ Low	IMPORTANT
**PSQI**												
2	Randomized trials	Serious^a^	Serious^b^	Not serious	Not serious	None^d^	82	81	–	MD 2.25 lower (3.88 lower to 0.61 lower)	⊕⊕○○ Low	IMPORTANT
**Peak VO_2_**												
2	Randomized trials	Serious^a^	Not serious	Not serious	Serious^c^	None^d^	23	23	–	MD 1.38 higher (1.51 lower to 4.28 higher)	⊕⊕○○ Low	IMPORTANT
**TUGT**												
2	Randomized trials	Serious^a^	Not serious	Not serious	Not serious	None^d^	55	55	–	MD 1.34 lower (2.5 lower to 0.19 lower)	⊕⊕⊕○ Moderate	IMPORTANT
**Heart failure hospitalization**												
3	Randomized trials	Serious^a^	Not serious	Not serious	Not serious	None^d^	12/120 (10.0%)	25/117 (21.4%)	RR 0.47 (0.25 to 0.88)	113 fewer per 1,000 (from 160 fewer to 26 fewer)	⊕⊕⊕○ Moderate	IMPORTANT

^a^The quality of majority of trials were not high. ^b^Unexplained heterogeneity. ^c^The small size or the combined effect size passing the invalid line. ^d^Funnel plots not completed due to<10 studies in the meta-analysis. BNP, B-type natriuretic peptide; CI, confidence interval; HAMD, Hamilton Depression Rating Scale; LVEF, left ventricular ejection fraction; MD, mean difference; MLHFQ, Minnesota Living with Heart Failure Questionnaire; NT-pro-BNP, N-terminal fragment of pro-BNP; Peak VO_2_, peak oxygen uptake; PSQI, Pittsburgh Sleep Quality Index; RR, risk ratio; SMD, standardized mean difference; TUGT, timed up and go test; 6MWT, 6-min walk test.

## Discussion

### Summary of evidence

This systematic review and meta-analysis included 15 RCTs that evaluated the effects of Tai Chi on health status among adults with CHF. Tai Chi significantly reduced MLHFQ scores, HAMD scores, PSQI scores, the time of TUGT, and the risk of heart failure hospitalization, favorably improved 6MWT, LVEF, and BNP/NT-pro-BNP compared with usual care controls. There was an improving trend for peak VO_2_ though the estimates failed to achieve statistical significance. There was no difference between the Tai Chi and the control group in reducing all-cause mortality or cardiovascular death. This might be due to the small sample size. No severe adverse events were observed in the patients treated with Tai Chi.

Minnesota Living with Heart Failure Questionnaire was a self-administered questionnaire consisting of 21 individual items. It provided an overall measure of health and summary scores of the physical and emotional dimensions of health (41). This heart failure-specific questionnaire has shown similar metric properties compared with other specific questionnaires widely used in clinical research such as the Kansas City Cardiomyopathy Questionnaire (42). Health-related quality of life was evaluated using MLHFQ (43). Higher scores indicated the poorer quality of life. In this meta-analysis, evidence suggested that Tai Chi effectively reduced MLHFQ scores, which indicated the improvement in quality of life.

The function limitation of exercise capacity was evaluated by 6MWT in accordance with American Thoracic Society guidelines (44), 6MWT measured the maximal distance an individual could walk in 6 min. Besides, the aerobic function was assessed using peak VO_2_. A previous study (45) demonstrated that a modest increase in peak VO_2_ over 3 months was associated with a more favorable outcome among patients with CHF. Monitoring the change in peak VO_2_ for such patients might have benefits in assessing prognosis. What is more, TUGT was performed to evaluate mobility and function in activities of daily living (46). Mobility was assessed using TUGT, in which the time to arise from a chair, walk 3 m, return 3 m, and sit down again was recorded (47). A shorter time indicated better mobility. This meta-analysis showed that Tai Chi effectively improved 6WMT and TUGT. The effects of Tai Chi on peak VO_2_ could not be evaluated due to insufficient data.

Cardiac function was assessed by LVEF and BNP/NT-pro-BNP. Compared with the usual care alone, Tai Chi plus the usual care was effective in improving LVEF and BNP/NT-pro-BNP. In the LVEF meta-analysis, the mean LVEF before treatment was greater than 50% in two studies (30, 37). Although improvement in LVEF may not be considered as an endpoint in patients with CHF with preserved EF at baseline, Tai Chi, as one of the means of cardiac rehabilitation, was widely used in clinical practice no matter what type of patients with HF. Also, in echocardiography, LVEF was an important indicator for evaluating cardiac function in patients. Among patients with HF, BNP was modestly associated with LVEF change. Therefore, all studies that reported the effect of Tai Chi on LVEF were included in the meta-analysis. However, in our meta-analysis, the certainty of evidence about LVEF and BNP/NT-pro-BNP ranged from very low to low due to the risk of bias, inconsistency, and publication bias. Although sensitivity analyses showed that the results were robust, the high heterogeneity suggested patients in the included studies received more comprehensive treatment besides Tai Chi, including dietary, health education, and pharmacologic therapy. Patients with more comprehensive intervention might show greater improvements both in LVEF and in BNP/NT-pro-BNP, which might exaggerate the effect of Tai Chi in improving cardiac function.

Depression symptom severity was evaluated with the 17-item HAMD (48). Sleep disturbance was measured by PSQI (49), and the total score range was 0 to 21. Higher scores indicated more severe depressive symptoms and worse sleep quality. This meta-analysis showed that Tai Chi effectively improved HAMD and PSQI.

Long-term cardiovascular prognosis after patients with CHF with Tai Chi was assessed by evaluating heart failure hospitalization, all-cause mortality or cardiovascular death, and adverse events. The evidence demonstrated that Tai Chi could reduce the risk of hospitalization and no adverse events were observed. However, the effects on all-cause mortality or cardiovascular death still need more data to evaluate.

In the subgroup analyses of this study, Tai Chi significantly improved MLHFQ scores, 6MWT, LVEF, and BNP/NT-pro-BNP on the 3- and 6-month courses with notable effects, which suggested that Tai Chi might have better effects during these courses. However, the heterogeneities were not eliminated, and differences remained among the studies in terms of the limited number of studies, sample size, different usual care controls, and concerns regarding the disease. Specifically, the high percentage of the studies applied usual pharmacologic therapy combined with other means of health care, suggesting that probably, it is not a particular Tai Chi effect, but some extra health care or physical activities, which provided improvement in quality of life and exercise tolerance above standard medical therapy. Thus, these results should be interpreted with caution.

### Clinical benefits and mechanism of Tai Chi

Tai Chi was regarded as a typical mind-body exercise combining aerobic exercise and meditation, which was beneficial for physical and mental health. Several potential mechanisms from physiological, psychological, and neurological perspectives might be related to the effect of Tai Chi (50). The possible links exited between the alterations in the brain and Tai Chi, including improved motor functions, pain perception, metabolic profile, cognitive functions, mental health, and sleep quality (51). Tai Chi could induce the modulation of brain morphology, functional homogeneity and connectivity, regional activity, and macro-scale network activity on health. One study (52) detected that long-term Tai Chi could improve motor function, especially gait and balance, in Parkinson’s disease. The underlying mechanisms might include enhanced brain network function, reduced inflammation, improved amino acid metabolism, energy metabolism, and neurotransmitter metabolism, and decreased vulnerability to dopaminergic degeneration. What is more, Tai Chi was also demonstrated the effect on preventing heart disease risk. Previous studies proved that Tai Chi could improve coronary heart disease prognosis by inactivating the mitogen-activated protein kinase pathway *via* serum miR-126 and affecting serum levels of the miR-24 and miR-155 (53, 54).

### Comparison to previous systematic review evidence

The effects of Tai Chi on MLHFQ, 6MWT, LVEF, and BNP/NT-pro-BNP detected were generally similar to previous studies (17, 20–23). However, one meta-analysis conducted by Ren et al. in 2017 (17) demonstrated inconclusive clinical evidence about Tai Chi in CHF. In this review, Tai Chi decreased the time of TUGT, which was different from Ren et al.’s results. In addition, we also conducted subgroup analyses, suggesting that the efficacy of Tai Chi might be related to the duration of treatment. Considering the limitations of Ren et al.’s study, we examined the study quality for each outcome and assessed the overall strength of the evidence taking into consideration the risk of bias, inconsistency, imprecision, indirectness of the evidence, and publication bias across each outcome, to rate the certainty of the evidence for each outcome. Besides, this review provided evidence on long-term clinical outcomes that were not available in Ren et al.’s study, including heart failure hospitalization, all-cause mortality or cardiovascular death, and adverse events. Not only that, we also added new outcomes HAMD and PSQI to assess the effects of Tai Chi on health status in patients with CHF comprehensively.

What is more, a recent systematic overview (24) evaluated the quality of existing systematic reviews and meta-analyses on Tai Chi for CHF. According to the study, despite positive results of all 6 of them, evaluation of Assessing the Methodological Quality of Systematic Reviews 2 (AMSTAR-2) and other tools for critical appraisal demonstrated an unsatisfactory quality of outcome measures. Compared with the six studies included in Huang et al.’s study (24), the protocol of this meta-analysis was previously registered in the PROSPERO. Also, this meta-analysis reported the list of 10 excluded studies and reason for their exclusion and analyzed the risk of bias for conflict of interests according to the funding sources in the included RCTs. To some extent, it improved the methodological quality and reporting quality and reduced the risk of bias of this systematic review and meta-analysis. In brief, this review provided a more comprehensive assessment of included studies to evaluate the effect of Tai Chi in patients with CHF.

### Limitations and strengths of this systematic review

#### Limitations

First, this systematic review could not comprehensively assess the health status of patients with CHF because of the limited outcome measures reported in the included studies. Second, because of the characteristic of Tai Chi, which was difficult to perform blinding, the non-high methodologic quality of evidence probably influenced the effect estimates. Moreover, participants who were interested in Tai Chi might have positive beliefs and expectations about the benefits of exercise, which might exaggerate the effect of Tai Chi and introduce biases in the outcomes. Third, significant heterogeneity remained in our meta-analysis, and the source of the heterogeneity could be associated with the wide variation of the frequency and duration of Tai Chi across studies, the different styles or forms of Tai Chi, different types of CHF, and different NYHA classes. Most noteworthy, there was a certain heterogeneity in terms of the controls due to the inconsistent interventions and high percentage of other health care in each RCT. This could lead to the results that overstate the effect of Tai Chi. But the subgroup analysis could not be conducted due to the insufficient numbers and the not high quality of included trials. Fourth, the duration of Tai Chi was relatively short, and even the longest duration was 12 months. Meanwhile, long-term prognosis of CHF with Tai Chi was less known. Finally, the included participants in this review were from China, Italy, and the United States. The universal applicability still could not be estimated in other races and regions.

#### Strengths

This meta-analysis had several strengths, including adherence to the guidelines of PRISMA, and the protocol was previously registered in the PROSPERO. What is more, the health status of CHF was the main issue of concern in this review. Besides the patient-reported quality of life, cardiac function, and exercise capacity, health status also includes mental health and sleep quality, which were associated with the prognosis and have an impact on the CHF patients’ quality of life. Furthermore, prognosis-related outcomes were measured in this meta-analysis, including heart failure hospitalization, all-cause mortality or cardiovascular death, and adverse events, which have not been concerned in the previous studies. Finally, we graded the quality of the body of evidence using the appropriate methodology (GRADE).

## Conclusion

In summary, compared with usual care alone, Tai Chi combined with usual care exhibited a specific advantage in improving health status of patients with CHF. Moreover, Tai Chi might show an effect based on a wide range of practice duration or “Tai Chi dose.” Considering the improvement in exercise capacity and quality of life, Tai Chi seemed more beneficial in the long term (6 months) than that in the short term (3 months). As for the effects on cardiac function and cardiovascular prognosis, the long-term clinical outcomes need to be further studied for the evaluation of Tai Chi’s clinical benefit. In terms of the limitations of the current research, mental health in patients with CHF should be regarded as a hot spot of special concern, which should be potentially considered in the future research. More multi-center, large-sample, high-quality RCTs with longer follow-up are required in the future to evaluate the effects of Tai Chi in patients with CHF and provide a more reliable reference for the clinic.

## Data availability statement

The original contributions presented in this study are included in the article/Supplementary material, further inquiries can be directed to the corresponding authors.

## Author contributions

FX and WC directed and supervised the study. JH and YW designed the protocol. JH, YW, and JZ were responsible for data collection and statistical analysis. JH wrote the first draft. JZ and WC reviewed and checked the manuscript. All authors have read and approved the final manuscript.
