# Supplementary material for: Effects of Tai Chi on health status in adults with chronic heart failure: A systematic review and meta-analysis
Source: Front Cardiovasc Med. 2022 Sep 9;9:953657. doi: 10.3389/fcvm.2022.953657 (PMC9500215; doi:10.3389/fcvm.2022.953657)
Supplement: Supplementary file 1 [file Data_Sheet_1.docx]

Table 1. PRISMA 2020 Checklist

| **Section and Topic** | **Item #** | **Checklist item** | **Location where item is reported** |
| --- | --- | --- | --- |
| **TITLE** | | |  |
| Title | 1 | Identify the report as a systematic review. | 1 |
| **ABSTRACT** | | |  |
| Abstract | 2 | See the PRISMA 2020 for Abstracts checklist. | 3, Supplementary Table 1. |
| **INTRODUCTION** | | |  |
| Rationale | 3 | Describe the rationale for the review in the context of existing knowledge. | 2 |
| Objectives | 4 | Provide an explicit statement of the objective(s) or question(s) the review addresses. | 2, 3 |
| **METHODS** | | |  |
| Eligibility criteria | 5 | Specify the inclusion and exclusion criteria for the review and how studies were grouped for the syntheses. | 4, 5 |
| Information sources | 6 | Specify all databases, registers, websites, organisations, reference lists and other sources searched or consulted to identify studies. Specify the date when each source was last searched or consulted. | 3 |
| Search strategy | 7 | Present the full search strategies for all databases, registers and websites, including any filters and limits used. | 4, Supplementary Table 2. |
| Selection process | 8 | Specify the methods used to decide whether a study met the inclusion criteria of the review, including how many reviewers screened each record and each report retrieved, whether they worked independently, and if applicable, details of automation tools used in the process. | 4, 5 |
| Data collection process | 9 | Specify the methods used to collect data from reports, including how many reviewers collected data from each report, whether they worked independently, any processes for obtaining or confirming data from study investigators, and if applicable, details of automation tools used in the process. | 4, 5 |
| Data items | 10a | List and define all outcomes for which data were sought. Specify whether all results that were compatible with each outcome domain in each study were sought (e.g. for all measures, time points, analyses), and if not, the methods used to decide which results to collect. | 4 |
|  | 10b | List and define all other variables for which data were sought (e.g. participant and intervention characteristics, funding sources). Describe any assumptions made about any missing or unclear information. | 4 |
| Study risk of bias assessment | 11 | Specify the methods used to assess risk of bias in the included studies, including details of the tool(s) used, how many reviewers assessed each study and whether they worked independently, and if applicable, details of automation tools used in the process. | 5 |
| Effect measures | 12 | Specify for each outcome the effect measure(s) (e.g. risk ratio, mean difference) used in the synthesis or presentation of results. | 5, 6 |
| Synthesis methods | 13a | Describe the processes used to decide which studies were eligible for each synthesis (e.g. tabulating the study intervention characteristics and comparing against the planned groups for each synthesis (item #5)). | 5, 6 |
|  | 13b | Describe any methods required to prepare the data for presentation or synthesis, such as handling of missing summary statistics, or data conversions. | 5, 6 |
|  | 13c | Describe any methods used to tabulate or visually display results of individual studies and syntheses. | 5, 6 |
|  | 13d | Describe any methods used to synthesize results and provide a rationale for the choice(s). If meta-analysis was performed, describe the model(s), method(s) to identify the presence and extent of statistical heterogeneity, and software package(s) used. | 5, 6 |
|  | 13e | Describe any methods used to explore possible causes of heterogeneity among study results (e.g. subgroup analysis, meta-regression). | 6 |
|  | 13f | Describe any sensitivity analyses conducted to assess robustness of the synthesized results. | 6 |
| Reporting bias assessment | 14 | Describe any methods used to assess risk of bias due to missing results in a synthesis (arising from reporting biases). | 6 |
| Certainty assessment | 15 | Describe any methods used to assess certainty (or confidence) in the body of evidence for an outcome. | 6 |
| **RESULTS** | | |  |
| Study selection | 16a | Describe the results of the search and selection process, from the number of records identified in the search to the number of studies included in the review, ideally using a flow diagram. | 7 |
|  | 16b | Cite studies that might appear to meet the inclusion criteria, but which were excluded, and explain why they were excluded. | 7, Supplementary Table 3. |
| Study characteristics | 17 | Cite each included study and present its characteristics. | 7, Table 1. |
| Risk of bias in studies | 18 | Present assessments of risk of bias for each included study. | 7, 8 |
| Results of individual studies | 19 | For all outcomes, present, for each study: (a) summary statistics for each group (where appropriate) and (b) an effect estimate and its precision (e.g. confidence/credible interval), ideally using structured tables or plots. | 24-26 |
| Results of syntheses | 20a | For each synthesis, briefly summarise the characteristics and risk of bias among contributing studies. | 8-11 |
|  | 20b | Present results of all statistical syntheses conducted. If meta-analysis was done, present for each the summary estimate and its precision (e.g. confidence/credible interval) and measures of statistical heterogeneity. If comparing groups, describe the direction of the effect. | 8-11 |
|  | 20c | Present results of all investigations of possible causes of heterogeneity among study results. | 8-11 |
|  | 20d | Present results of all sensitivity analyses conducted to assess the robustness of the synthesized results. | 8-11 |
| Reporting biases | 21 | Present assessments of risk of bias due to missing results (arising from reporting biases) for each synthesis assessed. | 11 |
| Certainty of evidence | 22 | Present assessments of certainty (or confidence) in the body of evidence for each outcome assessed. | 11, Table 3. |
| **DISCUSSION** | | |  |
| Discussion | 23a | Provide a general interpretation of the results in the context of other evidence. | 11, 12 |
|  | 23b | Discuss any limitations of the evidence included in the review. | 13-15 |
|  | 23c | Discuss any limitations of the review processes used. | 13-15 |
|  | 23d | Discuss implications of the results for practice, policy, and future research. | 15, 16 |
| **OTHER INFORMATION** | | |  |
| Registration and protocol | 24a | Provide registration information for the review, including register name and registration number, or state that the review was not registered. | 3 |
|  | 24b | Indicate where the review protocol can be accessed, or state that a protocol was not prepared. | 3 |
|  | 24c | Describe and explain any amendments to information provided at registration or in the protocol. | 3 |
| Support | 25 | Describe sources of financial or non-financial support for the review, and the role of the funders or sponsors in the review. | 16, 17 |
| Competing interests | 26 | Declare any competing interests of review authors. | 23 |
| Availability of data, code and other materials | 27 | Report which of the following are publicly available and where they can be found: template data collection forms; data extracted from included studies; data used for all analyses; analytic code; any other materials used in the review. | 16, Automatically attached at the end of the article after publication |

*From:*  Page MJ, McKenzie JE, Bossuyt PM, Boutron I, Hoffmann TC, Mulrow CD, et al. The PRISMA 2020 statement: an updated guideline for reporting systematic reviews. BMJ 2021;372:n71. doi: 10.1136/bmj.n71

For more information, visit: <http://www.prisma-statement.org/>

Table 2. Search strategies

| Databases | Terms |
| --- | --- |
| PubMed | #1 "Tai Ji"[MeSH Terms]  #2 "Tai-ji"[Title/Abstract] OR "tai chi"[Title/Abstract] OR "chi tai"[Title/Abstract] OR "tai ji quan"[Title/Abstract] OR "ji quan tai"[Title/Abstract] OR "quan tai ji"[Title/Abstract] OR "Taiji"[Title/Abstract] OR "Taijiquan"[Title/Abstract] OR "t ai chi"[Title/Abstract] OR "tai chi chuan"[Title/Abstract]  #3 #1 OR #2  #4 "Heart Failure"[MeSH Terms]  #5 "cardiac failure"[Title/Abstract] OR "heart decompensation"[Title/Abstract] OR "decompensation heart"[Title/Abstract] OR "myocardial failure"[Title/Abstract] OR "congestive heart failure"[Title/Abstract] OR "heart failure congestive"[Title/Abstract]  #6 #4 OR #5  #7 #3 AND #6 |
| Embase | #1 'tai chi'/exp  #2'tai ji':ab,ti OR 'chi, tai':ab,ti OR 'tai ji quan':ab,ti OR 'ji quan, tai':ab,ti OR 'quan, tai ji':ab,ti OR taiji:ab,ti OR taijiquan:ab,ti OR 'tai chi chuan':ab,ti  #3 'heart failure'/exp  #4'cardiac failure':ab,ti OR 'heart decompensation':ab,ti OR 'decompensation, heart':ab,ti OR 'myocardial failure':ab,ti OR 'congestive heart failure':ab,ti OR 'heart failure, congestive':ab,ti  #5 #1 OR #2  #6 #3 OR #4  #7 #5 AND #6 |
| The Cochrane Library | #1 MeSH descriptor: [Tai Ji] explode all trees  #2 ("Tai-ji" OR "Tai Chi" OR "Chi, Tai" OR "Tai Ji Quan" OR "Ji Quan, Tai" OR "Quan, Tai Ji" OR Taiji OR Taijiquan OR "Tai Chi Chuan" OR "T’ai Chi"):ti,ab,kw  #3 MeSH descriptor: [Heart Failure] explode all trees  #4 "Cardiac Failure" OR "Heart Decompensation" OR "Decompensation, Heart" OR "Myocardial Failure" OR "Congestive Heart Failure" OR "Heart Failure, Congestive"  #5 #1 OR #2  #6 #3 OR #4  #7 #5 AND #6 |
| Web of Science | #1 TS=(Tai Ji)  #2 (((((((((TI=(Tai-ji )) OR TI=(Tai Chi)) OR TI=(Chi, Tai)) OR TI=(Tai Ji Quan)) OR TI=(Ji Quan, Tai)) OR TI=(Quan, Tai Ji)) OR TI=(Taiji)) OR TI=(Taijiquan)) OR TI=(T’ai Chi)) OR TI=(Tai Chi Chuan)  #3 (((((((((AB=(Tai-ji)) OR AB=(Tai Chi)) OR AB=(Chi, Tai)) OR AB=(Tai Ji Quan)) OR AB=(Ji Quan, Tai)) OR AB=(Quan, Tai Ji)) OR AB=(Taiji)) OR AB=(Taijiquan)) OR AB=(T’ai Chi)) OR AB=(Tai Chi Chuan)  #4 TS=(Heart Failure)  #5 (((((TI=(Cardiac Failure)) OR TI=(Heart Decompensation)) OR TI=(Decompensation, Heart)) OR TI=(Myocardial Failure)) OR TI=(Congestive Heart Failure)) OR TI=(Heart Failure, Congestive)  #6 (((((AB=(Cardiac Failure)) OR AB=(Heart Decompensation)) OR AB=(Decompensation, Heart)) OR AB=(Myocardial Failure)) OR AB=(Congestive Heart Failure)) OR AB=(Heart Failure, Congestive)  #7 ((#1) OR #2) OR #3  #8 ((#4) OR #5) OR #6  #9 (#7) AND #8 |
| China National Knowledge Infrastructure | （主题：太极）AND（主题：心力衰竭 + 心衰 + 心功能不全） |
| Wanfang Database | 主题:(太极) and 主题:(心力衰竭 or 心衰 or 心功能不全) |
| Chinese Biomedical Database | "太极"[常用字段:智能] AND( "心力衰竭"[常用字段:智能] OR "心衰"[常用字段:智能] OR "心功能不全"[常用字段:智能]) |
| Chinese Scientific Journal Database | 题名或关键词=太极 AND 题名或关键词=心力衰竭 or 心衰 or 心功能不全 |

Table 3. Selected excluded studies and reason for their exclusion

| Study | Reason for exclusion |
| --- | --- |
| Redwine, L. S., et al. (2020). "An exploratory randomized sub-study of light-to-moderate intensity exercise on cognitive function, depression symptoms and inflammation in older adults with heart failure." Journal of psychosomatic research 128: 109883. | Outcomes irrelevant |
| Huang, C. (2014). "The study of Tai Chi bao qiu yun shou for Coronary Heart Disease, Chronic Heart Failure (NYHA grade Ⅲ) in patients with Cardiac Function recovery." Fujian University of Traditional Chinese Medicine. | Intervention period less than 3 months |
| Wang, Y. H., et al. (2019). "Effect of shadowboxing exercise combined with external counterpulsation on cardiac function and quality of life in patients with chronic heart failure." Modern Journal of Integrated Traditional Chinese and Western Medicine 28: 3901-3904. | Intervention period less than 3 months |
| Luberto, C. M., et al. (2020). "Exploring correlates of improved depression symptoms and quality of life following tai chi exercise for patients with heart failure." Esc Heart Failure 7(6): 4206-4212. | No extractable data |
| Redwine, L. S., et al. (2019). "A Randomized Study Examining the Effects of Mild-to-Moderate Group Exercises on Cardiovascular, Physical, and Psychological Well-being in Patients With Heart Failure." Journal of cardiopulmonary rehabilitation and prevention 39(6): 403-408. | No extractable data |
| Hagglund, L., et al. (2018). "A mixed methods study of Tai Chi exercise for patients with chronic heart failure aged 70 years and older." Nursing Open 5(2): 176-185. | No extractable data |
| Yeh, G. Y., et al. (2011). "Tai chi exercise in patients with chronic heart failure: a randomized clinical trial." Archives of internal medicine 171(8): 750-757. | No extractable data |
| Yeh, G. Y., et al. (2016). "Correlates of Exercise Self-efficacy in a Randomized Trial of Mind-Body Exercise in Patients With Chronic Heart Failure." Journal of cardiopulmonary rehabilitation and prevention 36(3): 186-194. | No extractable data |
| Yuan, L. H., et al. (2016). "Effect of Tai Chi on improvement of depression, sleeping quality and quality of life in elderly patients with chronic congestive heart failure complicated with depression." Guangxi Medical Journal 38: 1547-1550. | Duplicate data |
| Yeh, G. Y., et al. (2008). "T'ai Chi exercise in patients with chronic heart failure." Medicine and sport science 52: 195-208. | Duplicate data |

Table 4. Funding sources of the included 15 RCTs

| Author, year | Funding sources |
| --- | --- |
| Caminiti et al., 2011 | Not report |
| Ding and Chen, 2018 | Beijing Municipal Commission of Education Social Science program general project (SM201611417009) |
| Liu, 2017 | Not report |
| Pan, 2016 | Not report |
| Sang et al., 2015a | Project of Jilin Provincial Department of Health (No. 2011Z086) |
| Sang et al., 2015b | Project of Jilin Provincial Department of Health (No. 2011Z086) |
| Wang, 2011 | Not report |
| Yang et al., 2021 | Research project of Sichuan Provincial Health and Family Planning Commission (17PJ021) |
| Yao et al., 2010 | Not report |
| Yeh et al., 2004 | Unrestricted educational grants from the Bernard Osher Foundation and in part by the Beth Israel Deaconess Medical Center General Clinical Research Center grant (RR 01032) from the National Institutes of Health (NIH) |
| Yeh et al., 2013 | The National Center for Complementary and Alternative Medicine (Yeh, K23 AT00002624) and in part by the Beth Israel Deaconess Medical Center General Clinical Research Center grant (RR 01032) from the NIH. |
| Yu et al., 2019 | Project supported by Guangdong Administration of Traditional Chinese Medicine (No. 20161272) |
| Yu et al., 2020 | Natural Science Foundation of Shanghai (No.19ZR1455700), Science and Technology Research Project of Shanghai Songjiang District (No. 2017SJKJGG52), Shanghai Municipal Commission of Health and Family Planning (No.201640244) and Community Medicine and Health Management of Shanghai Association of Integrated Traditional Chinese and Western Medicine (No. 2016SH31) |
| Yuan, 2017 | Not report |
| Zhou et al., 2020 | Not report |

Table 5. Data on LVEF in the included 11 RCTs

| Study ID | LVEF (%) | | | | | |
| --- | --- | --- | --- | --- | --- | --- |
|  | The Tai Chi group | | | The control group | | |
|  | Before treatment | After treatment | Mean Change | Before treatment | After treatment | Mean Change |
| Liu 2017 | 44.99±0.55 | 53.77±0.78 | 8.78±0.78 | 44.82±0.63 | 49.39±0.69 | 4.57±0.69 |
| Pan 2016 | 32.4±7.2 | 36.3±9.3 | 3.9±8.45 | 33.1±7.4 | 33.9±7.7 | 0.8±7.55 |
| Sang 2015a | 36.4±3 | 56.2±3.8 | 19.8±3.47 | 37±2.8 | 41.8±3.7 | 4.8±3.34 |
| Sang 2015b | 35.4±3.1 | 55.4±3.3 | 20±3.2 | 36.5±2.6 | 42.8±3.3 | 6.3±3.01 |
| Yang 2021 | 51.81±3.15 | 52.43±2.82 | 0.62±3 | 50.95±2.69 | 51.99±2.52 | 1.04±2.61 |
| Yao 2010 | 30.85±9.78 | 48.63±9.37 | 17.78±9.58 | 30.22±9.32 | 39.62±7.28 | 9.4±8.49 |
| Yeh 2013 | 62±9 | 62±9 | 0±9 | 65±8 | 64±7 | -1±7.55 |
| Yu 2019 | 31.5±8.6/30.8±8.8 | 40.4±7.9/42.4±9.2 | 10.25±2.79 | 32.2±8.4 | 38.8±8.2 | 6.6±2.1 |
| Yu 2020 | 46.4±3.2 | 54.2±6.7 | 7.8±5.8 | 46.5±4.5 | 50.6±5.4 | 4.1±5.01 |
| Yuan 2017 | 42.54±3.02 | 61.76±2.21 | 19.22±2.71 | 43.35±3.78 | 53.94±2.76 | 10.59±3.39 |
| Zhou 2020 | 41.19±4.63 | 52.06±5.1 | 10.87±4.88 | 41.07±5.38 | 48.72±4.88 | 7.65±5.15 |

Note: Data are presented as mean ± SD.

Table 6. Data on BNP/NT-pro-BNP in the included 9 RCTs

| Study ID | BNP/NT-pro-BNP (pg/mL) | | | | | | Outcome, unit |
| --- | --- | --- | --- | --- | --- | --- | --- |
|  | The Tai Chi group | | | The control group | | |  |
|  | Before treatment | After treatment | Mean Change | Before treatment | After treatment | Mean Change |  |
| Caminiti 2011 | 136.4±31 | 99.7±22 | -36.7±8 | 134.5±28 | 111.7±24 | -22.8±7 | NT-pro-BNP, pg/mL |
| Pan 2016 | 432.0±57.0 | 223.0±29 | -209±49.37 | 431.0±56.0 | 404.0±49.0 | -27±52.85 | BNP, ng/L |
| Sang 2015b | 67.3±9.2 | 31.8±3.8 | -35.5±8.01 | 66.8±9.1 | 42.5±5.9 | -24.3±8 | BNP, not report |
| Yang 2021 | 917.47±202.62 | 876.02±81.1 | -41.45±176.63 | 923.11±223.07 | 932.23±155.85 | 9.12±198.2 | NT-pro-BNP, pg/mL |
| Yeh 2004 | 329±377 | 281±365 | -48±371.15 | 285±340 | 375±429 | 90±392.15 | BNP, pg/mL |
| Yeh 2013 | 98±85 | 98±116 | 0±104.024 | 72±85 | 107±99 | 35±92.8 | BNP, ng/mL |
| Yu 2019 | 325.5±133.4/334.5±137.0 | 161.5±80.2/138.7±63.2 | -179.9±116.5 | 314.5±121.7 | 191.2±80.6 | -123.3±107.2 | BNP, pg/mL |
| Yu 2020 | 462.7±35.8 | 259.3±10.6 | -203.4±31.85 | 460.3±36.8 | 312.7±34.5 | -147.6±35.71 | BNP, pg/mL |
| Yuan 2017 | 1689.59±536.87 | 938.06±412.56 | -751.53±486.77 | 1667.32±554.78 | 1106.45±446.37 | -560.87±509.3 | BNP, pg/mL |

Note: Data are presented as mean ± SD.
